# Effectiveness of a Prevention of Mother-to-Child HIV Transmission Programme in an Urban Hospital in Angola

**DOI:** 10.1371/journal.pone.0036381

**Published:** 2012-04-30

**Authors:** Cristina Lussiana, Sofia Vanda Lôa Clemente, Angelo Ghelardi, Magda Lonardi, Ivan Alejandro Pulido Tarquino, Marco Floridia

**Affiliations:** 1 Infectious Diseases Laboratory, Hospital Divina Providencia, Luanda, Angola; 2 Clinical Direction, Hospital Divina Providencia, Luanda, Angola; 3 Project Direction, Hospital Divina Providencia, Luanda, Angola; 4 Pediatric Department, Hospital Divina Providencia, Luanda, Angola; 5 Infectious Diseases Laboratory, Hospital Divina Providencia, Luanda, Angola; 6 Department of Therapeutic Research and Medicines Evaluation, Istituto Superiore di Sanità, Rome, Italy; Vanderbilt University, United States of America

## Abstract

**Background:**

Antiretroviral therapy is effective in reducing rates of mother-to child transmission of HIV to low levels in resource-limited contexts but the applicability and efficacy of these programs in the field are scarcely known. In order to explore such issues, we performed a descriptive study on retrospective data from hospital records of HIV-infected pregnant women who accessed in 2007–2010 the Luanda Municipal Hospital service for prevention of mother-to-child transmission (PMTCT). The main outcome measure was infant survival and HIV transmission. Our aim was to evaluate PMTCT programme in a local hospital setting in Africa.

**Results:**

Data for 104 pregnancies and 107 infants were analysed. Sixty-eight women (65.4%) had a first visit before or during pregnancy and received combination antiretroviral treatment (ART) in pregnancy. The remaining 36 women (34.6%) presented after delivery and received no ART during pregnancy. Across a median cohort follow-up time of 73 weeks, mortality among women with and without ART in pregnancy was 4.4% and 16.7%, respectively (death hazard ratio: 0.30, 95% CI 0.07–1.20, p = 0.089). The estimated rates of HIV transmission or death in the infants over a median follow up time of 74 weeks were 8.5% with maternal ART during pregnancy and 38.9% without maternal ART during pregnancy. Following adjustment for use of oral zidovudine in the newborn and exposure to maternal milk, no ART in pregnancy remained associated with a 5-fold higher infant risk of HIV transmission or death (adjusted odds ratio: 5.13, 95% CI: 1.31–20.15, p = 0.019).

**Conclusions:**

Among the women and infants adhering to the PMTCT programme, HIV transmission and mortality were low. However, many women presented too late for PMTCT, and about 20% of infants did not complete follow up. This suggests the need of targeted interventions that maintain the access of mothers and infants to prevention and care services for HIV.

## Introduction

Antiretroviral therapy (ART) has proved effective in reducing rates of mother-to child transmission (MTCT) of Human Immunodeficiency Virus (HIV) to very low levels not only in resource-rich countries but also in some resource-limited contexts [1]–[5]. Published cohort studies, however, have often considered only women entering prenatal care at selected sites, who have good compliance with treatment, and maintain a regular postnatal care. Clinical trials for the prevention of HIV transmission (PMTCT) have also shown very good results, but such findings should be evaluated considering the patient and site selection process, the frequently scheduled visits, and the adoption of study procedures that may facilitate therapeutic adherence and prolonged permanence in care.

It is only partially known to which extent such favourable results may also be obtained by local PMTCT programs, that several countries and municipalities have implemented. In such programs, care is usually provided at a hospital level, and usually depends on local resources, with no or limited external support from international agencies or other sources. When implementing a PMTCT program, local hospitals may adhere to World Health Organization (WHO) guidelines [6], endorse local guidelines issued by national authorities, or follow protocols from clinical trials. However, the applicability of these programs and their efficacy in the field are scarcely known, and there is limited information in this context on maternal and infant outcomes and on the level of program uptake, retention in care, and loss to follow up. In order to explore such issues, we retrospectively evaluated the records of a perinatal hospital service in Luanda, Angola, to evaluate the main outcomes among women and infants accessing a local program for prevention of HIV mother-to-child transmission.

## Materials and Methods

### Design, setting, and outcomes

The study is a retrospective analysis of mother and infant data from the hospital records of the perinatal and HIV PMTCT service of the Municipal Hospital Divina Providencia, a general population hospital situated in the urban area of Luanda, Angola. Eligible subjects were HIV-infected pregnant women who accessed the service between March 2007 and August 2010 and delivered live newborns. For those women with repeated pregnancies during the study period, only the last occurred was considered. Cut-off date for follow up was June 2011. The Ethic Committee of our institution approved the study design. Written informed consent was obtained from all the participants in this study.

Clinical HIV status was defined according to the WHO definition [6], and CD4 cell counts were measured by flow cytometry. Gestational age was determined on the basis of the last menstrual period, ultrasound biometry, or both. Start of treatment in pregnancy was referred to pregnancy week. Preterm delivery was defined as delivery before 37 completed weeks of gestation. Infant feeding was classified as replacement feeding, breastfeeding or mixed. Replacement feeding and breastfeeding were defined by exclusive assumption of either replacement feeding or breast milk, respectively, in the first six months. Mixed replacement/breastfeeding was defined by alternation of replacement feeding and breast milk in the first six months or by substitution of breastfeeding with formula before six completed months of life. In the newborns, diagnosis (positive tests after 18 months of life) or exclusion (negative tests before or after 18 months) of HIV infection required consistent results of two different rapid blood tests (Determine HIV 1/2, Unipath Limited, Inverness Medical, Bedford, UK; Uni-Gold HIV, Trinity Biotech, Bray, County Wicklow, Ireland), in two occasions at least three months apart. HIV testing for both mothers and infants was free of charge.

The main outcomes evaluated were infant survival, HIV transmission rate and maternal survival after delivery. The main variables considered as possible determinants of HIV transmission were ART during pregnancy, oral zidovudine in the newborn, and mode of infant feeding.

### Program description

Women accessing the HIV PMTCT and perinatal care service at the Luanda Divina Providencia hospital are managed according to standardized procedures. Women who become pregnant while on treatment usually continue the ongoing regimen, unless the evaluation of treatment suggests a significant risk of toxicity or teratogenicity. In women with no previous antiretroviral treatment, a CD4 cell count is performed, and antiretroviral treatment is started soon if CD4 counts are below 350/mm^3^ or if the women has WHO clinical HIV stage III or IV. Otherwise, treatment is started at the beginning of third trimester. The standard regimen for pregnant women is zidovudine plus lamivudine plus nevirapine, administered twice a day. All antiretroviral drugs are given free of charge directly to the women in an amount sufficient to cover the interval between subsequent pregnancy visits (usually two weeks). Tolerability of treatment is assessed monitoring (free of charge) haemoglobin (Hb), blood urea nitrogen (BUN), aspartate aminotransferase (AST) and alanine aminotransferase (ALT), usually on a monthly basis. Women in clinical HIV stage II or higher according to the WHO definition also receive cotrimoxazole, and if tuberculosis (TB) treatment is needed, ART is suspended and specific TB treatment is given. At delivery, women receive intravenous zidovudine. If the woman presents before or at delivery, the newborn receives oral zidovudine within 2 hours from delivery, continued for the first four weeks of life. Cotrimoxazole is given to all infants.

Replacement feeding is usually recommended as the preferred infant mode of feeding, but feeding options are evaluated on a single case basis, and a 6-month breastfeeding under antiretroviral treatment may be considered if formula feeding is not regarded as adequate according to *AFASS* criteria (acceptable, feasible, affordable, sustainable and safe). Replacement-feeding mothers receive free of charge 500 g of powder milk at every infant visit. After delivery, a CD4 count is performed in all women in order to evaluate the indication to antiretroviral treatment. Women already on ART with indication to treatment maintain ART, irrespective of mode of feeding. In women already on ART but with no personal indication to treatment, ART is discontinued if exclusive replacement feeding is the option selected, and otherwise continued until the end of breastfeeding (usually six months). In women with no previous ART presenting after delivery, treatment is started if needed, according to the above treatment recommendations criteria. Follow up of women and their infants takes place in the same health facility. HIV testing in the infants is performed at 9, 12 and 18 months, and infants are usually followed at regular intervals until 24 months of age (usually, with monthly visits).

### Statistical analysis

Demographic data were summarized with descriptive statistics. Quantitative data were compared by either Student's t-test or Mann-Whitney U-test, according to the characteristics of data distribution (normal or skewed, respectively). Categorical data were compared using the chi-square test or the Fisher test, as appropriate. Odds ratios (OR) and 95% confidence intervals [CI] in univariate analyses were calculated by Mantel-Haenszel estimates.

The variables with a potential association with the main outcome (HIV transmission or death) in univariate analysis (p<0.10) were included in a multivariable logistic regression model which used occurrence of the main outcome as the dependent variable, calculating an adjusted odds ratio [AOR] with 95% CI for HIV transmission or death. Survival analyses were based on Kaplan-Meyer analysis, log-rank test, and Cox regression. Significance levels were set at 0.05. All the analyses were performed using the SPSS software, version 17.0 (SPSS Inc., Chicago, IL, US).

## Results

### Population

**Figure 1:**
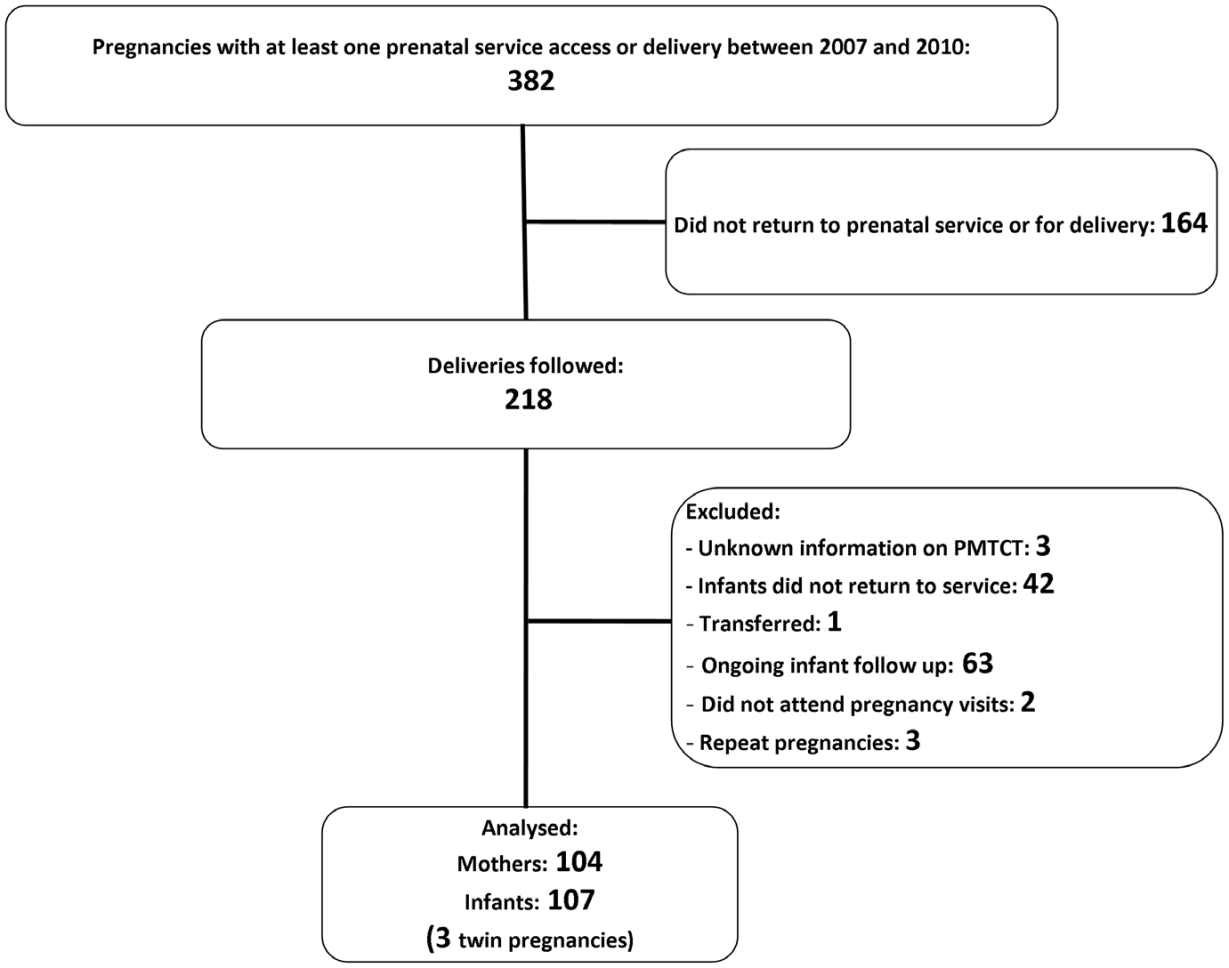
Population flowchart.

Among 382 women with at least one prenatal or perinatal service access, 218 (57.1%) were followed for delivery. Among their infants, 42/218 (19.3%) did not return to the hospital for follow up evaluations. Following exclusion of 63 infants with ongoing but limited follow up (with respect to HIV diagnosis), one transferred infant, three cases with unknown information on PMTCT, two cases not attending pregnancy visits and three repeated pregnancies between 2007 and 2010 in the same women, 104 mothers and 107 infants were available for subsequent analyses (Figure 1). Their general characteristics are shown in Table 1.

**Table 1:** General characteristics.

	N	Mean	Median	Interquartile range
Age (years):	104	29.2	29.0	25.0–33.0
Body mass index (kg/m^2^):	102	23.1	22.2	20.3–25.5
Number of previous pregnancies:	104	2.9	2.0	1.0–4.0
Number of previous live births:	104	2.5	2.0	1.0–3.7
Week of first visit in pregnancy:	68	16.7	16.0	10.2–22.0
Week of start of ART:	68	14.8	17.0	1.0–22.7
CD4 at first visit in pregnancy (cells/mm^3^):	68	372	359	224–486
			N	%
Primiparous (n: 104):			8	7.7
HIV status of the partner (n: 104):	HIV negative		26	25.0
	HIV positive		47	45.2
	Unknown		31	29.8
HBsAg positive (n: 99):			15	14.4
TB coinfected (n: 104):			9	8.7
WHO HIV disease stage (n: 103):	I		50	48.5
	II		32	31.1
	III		18	17.5
	IV		3	2.9
HIV infection diagnosed before current pregnancy (n: 104):			49	47.1
CD4 count <350 cells/mm^3^ at first visit in pregnancy (n: 68):			33	48.5
Antiretroviral treatment before pregnancy (n: 104):			20	19.2
First maternal visit (n:104):	before current pregnancy		29	27.9
	during current pregnancy		39	37.5
	at or after delivery		36	34.6
Trimester of first maternal visit in pregnancy (n: 68#):	first		27	39.7
	second		34	50.0
	third		7	10.3
Maternal ART in pregnancy#:	overall		68/104	65.4
	discontinued after delivery		21/68	30.1
	continued during follow up		47/68	69.9
No maternal ART in pregnancy:	overall		36/104	34.6
	ART started after delivery		17/36	47.2
	no ART before or after pregnancy		19/36	52.8

ART: antiretroviral treatment; TB: tuberculosis.

# for women who presented before delivery. Regimens: zidovudine/lamivudine/nevirapine: 62; zidovudine/lamivudine/efavirenz: 2; stavudine/lamivudine/nevirapine: 2; stavudine/lamivudine/efavirenz: 1; lamivudine/tenofovir/lopinavir: 1;

### Personal and pregnancy history

Most of the women (96/104, 92.3%) were previously pregnant at least once (average parity: 2.5). About half of the women (55/104, 52.9%), however, were not diagnosed with HIV before current pregnancy, and only 20 (19.2%) had a previous history of ART. Overall, 21 women (20.4%) were in WHO disease stage 3 or 4. HIV status was unknown for 31 partners (29.8%). Most of the partners with known HIV status (47/73, 64.4%) were HIV-infected. First maternal visit occurred before or during pregnancy for 68 women (65.4%). Among these women, 61 (89.7%) presented before the end of second trimester (Table 1).

### Pregnancy duration and delivery

Data on pregnancy duration was available for 69 women (66.3%). Preterm delivery involved 22/69 women (31.9%). Most of the women delivered at the hospital (hospital: 61/104, 58.7%; home: 11, 10.6%; unknown: 31, 29.8%, other: 1, 1.0%). All the women with recorded information on mode of delivery (n: 72, 69.2%) delivered vaginally. The main maternal and infant outcomes are reported in Table 2.

**Table 2:** Follow up and outcomes.

		Overall		ART during pregnancy		No ART during pregnancy	
		N	Median (IQR)	N	Median (IQR)	N	Median (IQR)
Week of change of ART:		7	24.0 (18.0–31.0)	7	24.0 (18.0–31.0)	-	-
Week of delivery:		69	37.0 (36.0–39.0)	68	37.5 (36.0–39.0)	1	28
Birthweight (g)*:		78	3000 (2600–3400)	62	2900 (2575–3300)	16	3400 (2762.5–3825)
First CD4 count following delivery (cells/mm^3^):		101	448 (279–657)	68	493.5 (345–699.5)	33	322 (199–458.5)
Maternal follow up post-delivery (weeks):		104	73.0 (54.0–94.7)	68	61.5 (53–77)	36	109 (78.75–145.75)
Infant follow up (weeks):		107*	74.0 (55.0–99.0)	71	64 (54–79)	36	123.5 (90.25–148.5)
		N	%	N	%	N	%
Preterm delivery:		22/69	31.9	21/68	30.9	1/1	100%
Infant feeding*:	Exclusive breastfeeding	36/107	33.6	11/71	15.5	25/36	69.4
	Exclusive formula feeding	62/107	58.0	57/71	80.3	5/36	13.9
	Mixed	9/107	8.4	3/71	4.2	6/36	16.7
Maternal breastfeeding beyond 6 months:		25/105	23.8	4/71	5.6	21/34	61.8
Maternal ART in the first 6 months after delivery:		51/104	49.0	49/68	72.1	2/36	5.6
Zidovudine to the newborn*:		57/107	53.3	56/71	78.9	1/36	2.8
Cotrimoxazole to the newborn*:		98/107	91.6	69/71	97.2	29/36	80.6
Maternal survival at the end of follow up:	Overall	95/104	91.3	65/68	95.6	30/36	83.3
	ART during and after pregnancy	-	-	44/47	93.6	-	-
	ART only after pregnancy	-	-	-	-	17/17	100.0
	ART only during pregnancy	-	-	21/21	100.0	-	-
	No ART before or after pregnancy	-	-	-	-	13/19	68.4
Infant status at the end of follow up*:	HIV-infected, deceased	4/107	3.7	1/71	1.4	3/36	8.3
	HIV-unknown, deceased	5/107	4.7	5/71	7.0	-	-
	HIV-unknown, lost to follow up	1/107	0.9	-	-	1/36	2.8
	HIV-infected, alive	10/107	9.3	-	-	10/36	27.8
	HIV-uninfected, alive	87/107	81.3	65/71	91.5	22/36	61.1

IQR: Interquartile range. ART: antiretroviral treatment.

* includes twins.

### Women who presented before or during pregnancy

For the 68 women with ART during pregnancy (including women already on treatment at conception), median time of first visit was week 16 and median week of first antiretroviral treatment in pregnancy was week 17. The most common antiretroviral regimen in pregnancy was lamivudine plus zidovudine plus nevirapine (62/68, 91.2%). All regimens included a backbone of two nucleosides (zidovudine-lamivudine: 64, stavudine-lamivudine: 3, tenofovir-lamivudine: 1), plus nevirapine (n: 64), efavirenz (n: 3) or lopinavir/ritonavir (n: 1). Only a few treatment changes occurred during pregnancy (n: 7, 10.3%), because of drug intolerance (n:2) and lack of therapeutic response (n: 5, defined by a CD4 count drop >25%, a viral load increase >30%, or development of opportunistic infections). Among 62 women with available information, 40 (64.5%) received intrapartum zidovudine. Among the 71 infants from mothers with ART in pregnancy, 14 (19.7%) received breast milk, through exclusive (11/71, 15.5%) or nonexclusive (3/71, 4.2%) breastfeeding. Eleven of their mothers (78.6%) were receiving ART during exclusive or nonexclusive breastfeeding,

### Women who presented post-delivery

Thirty-six women (34.6%) presented after delivery and received no ART during pregnancy. Most of their infants (31/36, 86.1%) received maternal milk, through exclusive (25/36, 69.4%) or nonexclusive (6/36, 16.7%) breastfeeding. In this group only one infant (2.8%) received oral zidovudine and only one mother (2.8%) received ART during breastfeeding. Overall, among these mothers who presented after delivery, 17 (47.2%) subsequently started ART because of low CD4 counts or concomitant opportunistic infections.

### Maternal follow up and survival

**Figure 2:**
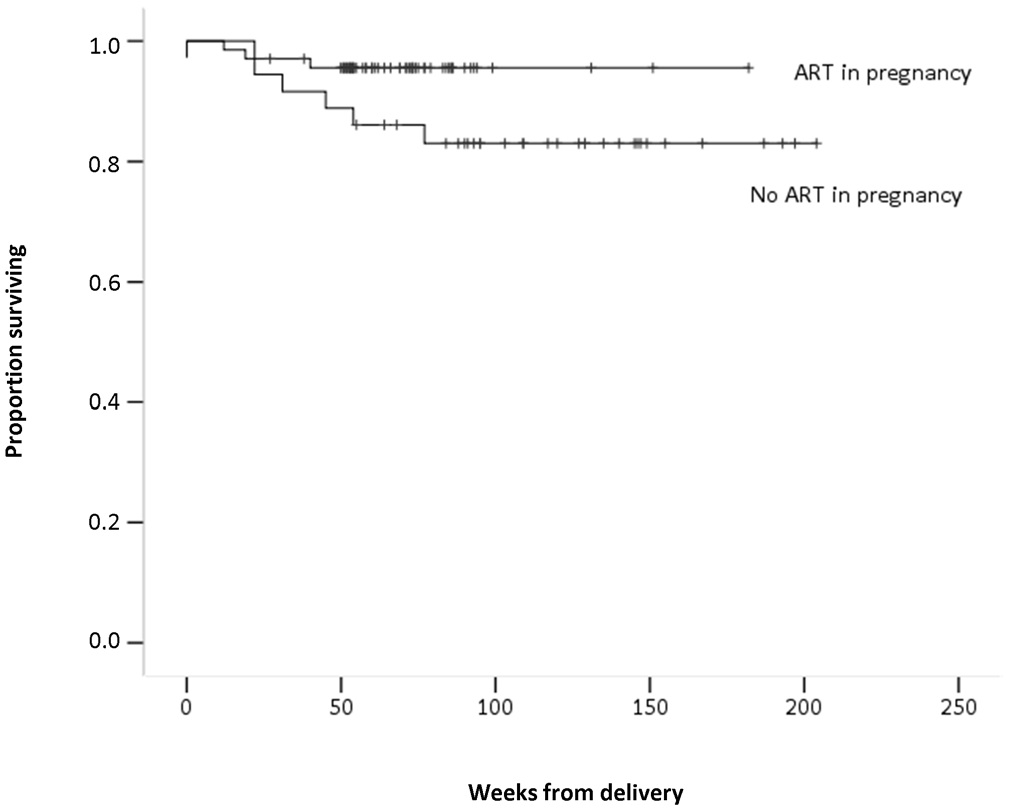
Kaplan-Meier Curves for maternal survival with and without ART in pregnancy.

Cumulative maternal follow up was 159.2 patient-years. Mean and median maternal follow up after delivery were 82.3 and 73 weeks, respectively. About half of the mothers (n: 51, 49.0%) received ART in the first six months after delivery. Nine mothers (8.7%) deceased during follow up (reasons unknown). Mortality over the follow up was 4.4% (3/68) among women with ART in pregnancy and 16.7% (6/36) among women with no ART in pregnancy. Survival probabilities at 96 weeks in a Kaplan-Meier analysis were 0.95 (95%CI 0.90–1.00) for women with ART in pregnancy and 0.83 (95%CI 0.70–0.96) for women with no ART in pregnancy (death hazard ratio: 0.30, 95% CI 0.07–1.20, p = 0.089) (Figure 2). Median values of last CD4 count in women with and without survival were 491 (n: 95) and 642 (n: 6), respectively (p = 0.179).

### Infants' follow up information

Three of the 104 pregnancies were twin pregnancies, for a total of 107 infants (male 57, female 50). Cumulative infant follow up was was 166.0 patient-years. Mean and median infant follow up were 86.4 and 74.0 weeks, respectively. Infant mode of feeding was represented by breastfeeding only (n: 36, 33.6%), replacement feeding only (n: 62, 58.0%) and mixed replacement/formula feeding (n: 9, 8.4%).

About one quarter of infants with available information (23.8%) received maternal milk for more than six months. Roughly half of the infants received oral zidovudine (n: 57, 53.3%). Overall, use of antiretroviral treatment in pregnancy was strongly associated with subsequent replacement feeding (odds ratio: 25.2, 95% CI 8.3–76.6, p<0.001), ART in the first six months after delivery (odds ratio: 43.3, 95% CI 9.5–197.6, p<0.001), and oral zidovudine in the newborn (odds ratio: 130.7, 95% CI 16.5–1033.2, p<0.001).

### Infant survival

**Figure 3:**
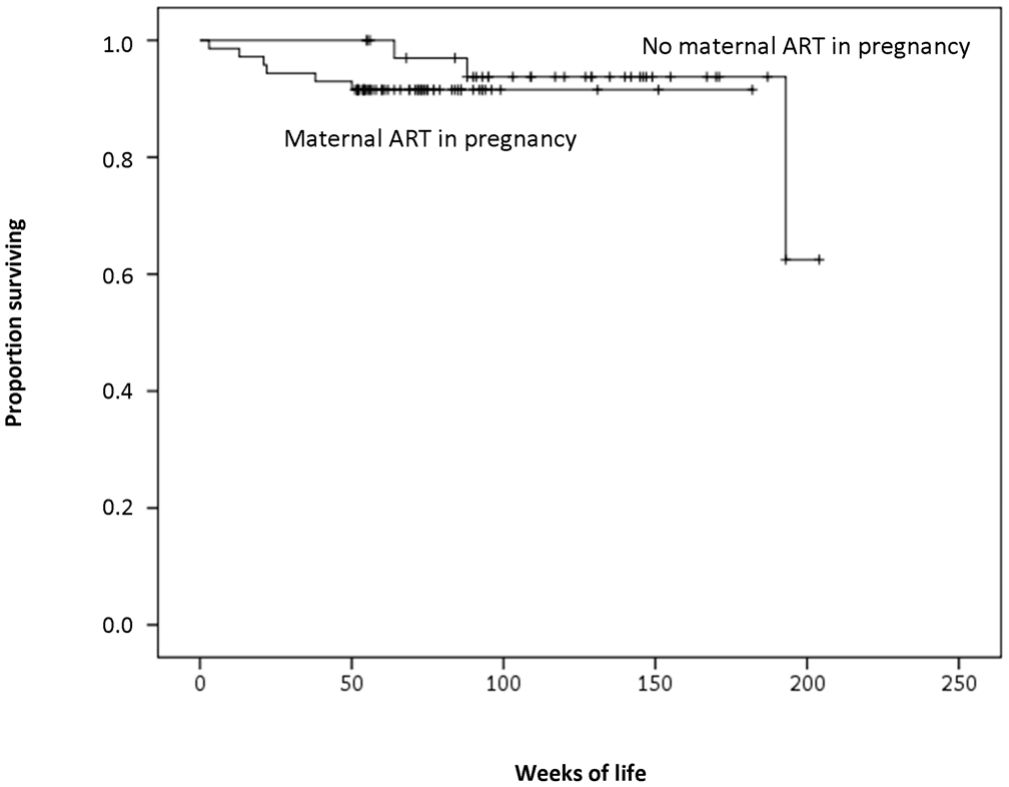
Kaplan-Meier Curves for infant survival with and without maternal ART in pregnancy.

Among the infants followed, nine (8.4%) deceased, five (4.7%) with an unknown HIV status, and four (3.7%) with confirmed HIV infection. Overall, 97 infants were alive at last follow up, with ten of them (10.3%) infected. One infant in the group with no maternal ART was lost to follow up, following an undetermined HIV test result. In the two groups of infants from mothers with (n: 71) and without (n: 36) ART during pregnancy, mortality was 8.5% and 8.3%, respectively. Survival probabilities at 96 weeks in a Kaplan-Meier analysis were 0.92 (95%CI 0.85–0.99) for infants from women with ART in pregnancy and 0.94 (95%CI 0.86–1.00) for infants from women with no ART in pregnancy (death hazard ratio: 2.25, 95%CI 0.42–11.96, p = 0.343) (Figure 3).

### HIV transmission

Considering infants with an ascertained HIV status only (n: 101), overall rate of HIV transmission was 13.1% (14/101); HIV transmission in the two groups of infants from mothers with (n: 66) and without (n: 35) ART during pregnancy were 1.5% (1/66) and 37.1% (13/35) (odds ratio for HIV transmission: 38.4, 95%CI 4.8–310.7, p = 0.001).

### HIV transmission or death

Considering infants with known HIV or survival status only (n: 102), overall, rate of HIV transmission or death was 14.7% (15/102). Assuming as a worst scenario HIV transmission for all cases with missing information on HIV status or survival, rate of HIV transmission or death would be 18.7% (20/107) for the entire group, 8.5% (6/71) among infants from mothers with ART during pregnancy and 38.9% (14/36) among infants from mothers with no ART during pregnancy (odds ratio: 6.89, 95%CI 2.36–20.1, p<0.001).

### Role of type of feeding and of neonatal zidovudine prophylaxis

Overall, HIV transmission or death by type of feeding was 36.1% (13/36) with exclusive breastfeeding only, 22.2% (2/9) with mixed feeding, and 8.1% (5/62) with exclusive replacement feeding (p = 0.003, chi-square test). The corresponding figures according to presence or absence of postpartum ART were 43.8% (7/16), 25.0% (1/4) and 9.5% (2/21) with no postpartum ART and 30.0% (6/20), 20.0% (1/5) and 7.3% (3/41) in the presence of postpartum ART. Overall, exposure to maternal milk (mixed or exclusive breastfeeding) was associated with a significantly higher risk of HIV transmission or death (unadjusted odds ratio: 5.70, 95%CI 1.89–17.2, p = 0.002). Finally, the risk of HIV transmission or death was significantly higher among infants with no zidovudine postnatal prophylaxis (16/50, 32.0%), compared to infants with postnatal zidovudine (4/57, 7.0%; unadjusted odds ratio: 6.23, 95% CI 1.92–20.23, p = 0.002).

### Multivariable analysis of HIV transmission and death

In a multivariable analysis that used as covariates ART during pregnancy, oral zidovudine in the newborn, and exposure to maternal milk (exclusive or mixed breastfeeding) in the first six months, no antiretroviral treatment in pregnancy remained associated with a higher risk of HIV transmission or death (adjusted odds ratio: 5.13, 95% CI: 1.31–20.15, p = 0.019). Adjusted odds ratios for use of oral zidovudine and exposure to maternal milk were 2.65 (95%CI 0.51–13.7, p = 0.244) and 1.25 (95%CI 0.64–2.46, p = 0.509), respectively.

### Tolerability of antiretroviral treatment during pregnancy

In women on antiretroviral treatment during pregnancy, the monitoring of haemoglobin (Hb), blood urea nitrogen (BUN), aspartate aminotransferase (AST) and alanine aminotransferase (ALT) showed no significant changes during pregnancy (data not shown). The occurrence of anaemia in this population was high, but did not show an increase with the advancing of pregnancy: overall, the proportion of women with haemoglobin values lower than 11 g/dl was 82.5% at first trimester and 70.6% at third trimester. Occurrence of increased levels of AST or ALT was infrequent: no woman had ALT or AST levels above 5 times the upper normal limit at any trimester of pregnancy, and only 6 women (9.7%) had during pregnancy a value between two and five times the upper normal limit.

## Discussion

MTCT of HIV is responsible for the vast majority of HIV paediatric cases, and represents a substantial source of paediatric mortality in countries with high HIV prevalence [7]. Interventions have proved effective in reducing rates of HIV transmission, even in resource-limited settings (RLS) [1]–[5], and current evidence from clinical trials and observational studies is periodically reviewed and translated into recommendations from WHO and other sources [8]–[9]. There is currently a widespread effort aimed at setting up effective local programs against MTCT, and the impact of some of these programs has been analysed [2], [4], [10]–[15].

Our study adds information to this context, describing the efficacy of a local PMTCT programme at an urban hospital in Angola. A first finding of our study is that a presentation to care and initiation of antiretroviral treatment in pregnancy may be associated with a better maternal survival following delivery: during a median cohort follow up time of 73 weeks, mortality rates were 4.4% and 16.7%, respectively, in women with and without antiretroviral treatment in pregnancy. Although this difference did not reach statistical significance in a survival analysis, the numbers were small, and a larger sample might have captured a significant effect. There is substantial evidence that antiretroviral treatment is associated with improved survival, and recent data collected in low income countries even suggest that persons receiving combination antiretroviral treatment can reach an almost normal life expectancy [16]. Our data are consistent with a positive effect of combination treatment in pregnant women that extends beyond delivery, translating in a better survival, as already reported by others [14], and indirectly reinforce the concept of using pregnancy as an important point for HIV testing and for entry into treatment and care.

In our population, the impact of HIV testing in pregnancy for entry into care was relevant, because a significant proportion of the women (52.9%) were unaware of being HIV-infected prior to accessing the hospital service, and most of them maintained antiretroviral treatment following delivery. It has also to be considered that starting antiretroviral treatment in women with a recent HIV diagnosis is likely to translate, in a context of serodiscordant couples, into a significantly lower risk of sexual HIV transmission to the uninfected partners, as recently demonstrated [17].

Entry of care before delivery was also associated with other positive interventions against HIV transmission, such as formula feeding (according to the AFASS criteria) and oral zidovudine prophylaxis in the newborn: women on antenatal ART had significantly higher probabilities of a subsequent replacement feeding (79.4% in this group) and oral zidovudine prophylaxis in the newborn (78.9%).

The benefits for the infants were straightforward: following adjustment for oral neonatal zidovudine and exposure to maternal milk, infants from women with no antiretroviral treatment in pregnancy had a five-fold increase in the risk of HIV transmission or death, for a rate of HIV transmission or death of 38.9% compared to the 8.5% rate observed among infants from women with ART in pregnancy. Considering infants with an ascertained HIV status only, we observed a remarkably low rate of HIV transmission (1.5%), but this rate excludes infants who died before having their HIV status defined. Cause of death for such infants was not reported and we could not therefore exclude fatal HIV-related events in the infants who could not be tested. Because of this possibility, we preferred to assume HIV transmission for all the infants deceased (n: 5) or lost to follow up with missing HIV status information (n: 1).

In terms of infant outcomes, it is expected that between 20% and 40% of infants born from HIV-infected mothers will eventually become infected with HIV in the absence of any intervention.

Local PMTCT programs implemented in resource-limited settings, despite some differences in the regimens used and in the context of care, have shown significant reductions in HIV transmission and mortality, with rates of transmission well below the above values, particularly when highly active antiretroviral therapy (HAART) was used [2].

Ekouevi et al. [1] have reported in Cote d'Ivoire a significantly lower transmission rate among infants with maternal HAART (2.3%), compared to single dose nevirapine (16.1%), and In two other PMTCT programs in South Africa and Cameroon, rates of HIV transmission of 5% [13] and 6.6% [11] were observed. In a study conducted in 2006–2008 in rural Kenya, occurrence of MTCT was more frequent (15%), although infant mortality was relatively limited [15]. The higher transmission rate in this study might reflect differences in health settings or social conditions, and might also be a consequence of the earlier diagnosis of infant infection with virological testing.

Other studies have also reported information on infant mortality: Kouanda et al. in urban Burkina Faso report a remarkable null transmission rate with maternal HAART, but infant mortality was present, even in the subgroup of maternal HAART [4]. In a PMTCT program conducted in Northern Uganda, the rate of HIV transmission or HIV-related death among infants was 15.5% [12].

Differences in social conditions and context of care are likely to greatly influence HIV transmission rate. Even if our local PMTCT programme may differ from other and often wider programmes described in the literature, our findings are fully consistent with the important reductions in HIV transmission obtained by other HAART-based PMTCT programmes: the HIV-free infant survival with maternal HAART observed in this study (91.5% at about 18 months of follow up) is substantially identical to the rate described within the larger, international-based DREAM programme (92.5% at 12 months) [2], and confirms in a different setting that infants from women attending local hospital PMTCT programmes may expect a substantially better outcome compared to the offspring of mothers not accessing PMTCT services. Our finding of similar survival probabilities in infants from mothers with and without ART in pregnancy highlight the importance of a regular follow up infant care, that might be at least as important (and possibly more important) than maternal ART in pregnancy.

Among the women who received ART in pregnancy, treatment was well tolerated, with no major complications. Anemia (Hb <11 g/dl) involved more than two thirds of the women on ART at third trimester. Similarly high rates have however been observed in the general population of pregnant women in other African countries, such as Ghana (54.0%) and southern Benin (65.7%), suggesting that this condition is quite common even in the absence of ART, possibly because of concomitant cofactors [18], [19]. In addition, the moderate decrease observed in the rate of anemia between first and third trimester suggests the safety of ART in pregnancy, with no significant worsening of hemoglobin concentrations even in a background of common maternal anemia. Preterm delivery was relatively common, but the information on duration of pregnancy was not available for all the women, and we cannot therefore exclude the possibility of a selection bias.

Our study has some limitations. First, the findings may be more easily applicable to urban contexts and less representative of rural settings, where different conditions may be found. We have also no information on those women that did not access antenatal care services and therefore were not reached by the existing programme. We also were unable to analyze precisely the impact of time on ART on transmission, because the exact date of start of ART was not available for all women.

Another important limitation is represented by the frequent loss to follow up. This limitation is however quite common in PMTCT programs in restrained-resources settings, and other studies have clearly shown the need to improve tracking of HIV-exposed infants [12], prophylaxis uptake, partner testing, and referral for treatment [10].

In our study, the proportion of women who after a first health access did not return for delivery or PMTCT was about 40%, and almost 20% of the infants from HIV-infected mothers did not return for follow up evaluations. Even if these numbers are concerning, an even lower coverage has been reported in similar programmes: Azcoaga-Lorenzo et al. report a 40.4% coverage for their programme in rural Kenya^15^, and in the Ethiopian study by Mirkuzie et al. only 10.6% of the positive women completed their follow up to child HIV testing [10].

It is not possible to define to which extent the exclusion of women lost to follow up and unable to complete the program may have led to an underestimation of HIV transmission and infant mortality. Nonetheless, our data on the outcomes of women and infants who delivered and followed-up in a local PMTCT program indicate clearly that accessing and following the PMTCT programmes is associated with significant individual benefits in terms of HIV transmission and survival. Those data emphasize the need to recognize and overcome existing barriers to access and permanence in care in order to improve the program efficacy on a population basis.

Some studies have addressed this issue: Duff et al. have identified a major role of economic concerns among women accessing an antenatal care clinic in Western Uganda: cost of transportation represented a major barrier to accessing treatment, together with HIV-related stigma, non-disclosure of HIV status, long waiting times and suboptimal provider-patient interactions at the clinic [20]. Lack of support from partners and negative community reactions were other relevant factors associated with drop out from a PMTCT program in Malawi [21].

In resource limited settings, the prevention of HIV vertical transmission seems to be adequately effective only if accompanied by an integrated approach that takes into account patients' social conditions and local difficulties. Integrating PMTCT with other services may improve linking to care and reduce the attrition rate, as suggested by the limited loss to follow up reported by programmes with a comprehensive treatment of HIV, malnutrition, and other concomitant diseases [2]. Involving communities is also an important component, and might facilitate tracking of infants, treatment uptake, and individual support. Education has also a key role, because higher levels of knowledge about HIV and PMTCT are associated with referral for testing [22], and might promote early diagnosis, and initiation of HAART before onset of symptomatic disease [20]. Assessment of gender issues, economical empowerment of the women, minimization of stigma and support from the community are also important [21]. In general, social conditions influence dramatically the outcome of preventive efforts against HIV transmission. Therefore, individually effective biomedical interventions, including antiretroviral therapy for PMTCT, will not be effective at a population level without concomitant interventions on the social environment that include education, women empowerment, minimization of stigma, accessibility of care and community involvement.
